# Short-term blood pressure variability is inversely related to regional amplitude of low frequency fluctuations in older and younger adults

**DOI:** 10.1016/j.nbas.2023.100085

**Published:** 2023-07-11

**Authors:** Isabel J. Sible, Hyun Joo Yoo, Jungwon Min, Kaoru Nashiro, Catie Chang, Daniel A. Nation, Mara Mather

**Affiliations:** a Department of Psychology, University of Southern California, Los Angeles, CA 90089, USA; b Davis School of Gerontology, University of Southern California, Los Angeles, CA 90089, USA; c Department of Electrical and Computer Engineering, Vanderbilt University, Nashville, TN 37235, USA; d Institute for Memory Impairments and Neurological Disorders, University of California, Irvine, Irvine, CA 92697, USA; e Department of Psychological Science, University of California Irvine, Irvine, CA 92697, USA; f Department of Biomedical Engineering, University of Southern California, Los Angeles, CA 90089, USA

**Keywords:** Blood pressure variability, Amplitude of low frequency fluctuations, Aging, Medial temporal lobes

## Abstract

Blood pressure variability (BPV), independent of mean blood pressure levels, is associated with cerebrovascular disease burden on MRI and postmortem evaluation. However, less is known about relationships with markers of cerebrovascular dysfunction, such as diminished spontaneous brain activity as measured by the amplitude of low frequency fluctuations (ALFF), especially in brain regions with vascular and neuronal vulnerability in aging. We investigated the relationship between short-term BPV and concurrent regional ALFF from resting state fMRI in a sample of community-dwelling older adults (*n* = 44) and healthy younger adults (*n* = 49). In older adults, elevated systolic BPV was associated with lower ALFF in widespread medial temporal regions and the anterior cingulate cortex. Higher systolic BPV in younger adults was also related to lower ALFF in the medial temporal lobe, albeit in fewer subregions, and the amygdala. There were no significant associations between systolic BPV and ALFF across the right/left whole brain or in the insular cortex in either group. Findings suggest a possible regional vulnerability to cerebrovascular dysfunction and short-term fluctuations in blood pressure. BPV may be an understudied risk factor for cerebrovascular changes in aging.

## Graphical abstract

**Figure:**
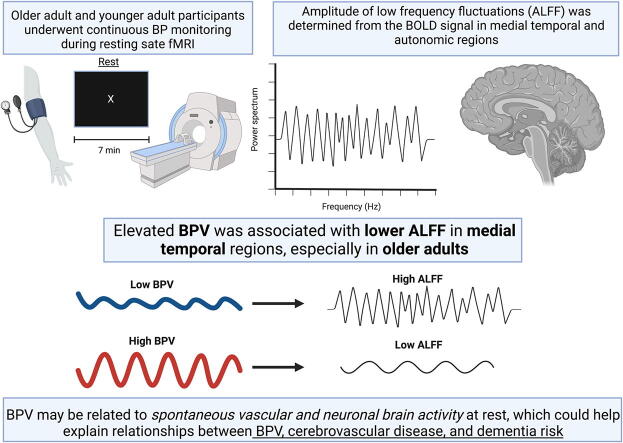

## 1 Introduction

Blood pressure (BP) control remains a promising therapeutic target for reducing risk of stroke, cerebrovascular disease, and dementia [17], [69]. In addition to the goal of lowering mean BP levels, newer evidence suggests BP variation over seconds, days, months, and years is also associated with deleterious cognitive and brain health outcomes, especially in older adults [32]. Interestingly, some studies indicate the predictive value of BP variability (BPV) exceeds that of mean BP levels for cerebrovascular disease burden, cognitive impairment, and dementia [8], [25].

While BPV has been associated with cerebrovascular disease on MRI [26], [63] and postmortem evaluation [25], [48], less is known about relationships with cerebrovascular dysfunction that may precede frank markers of cerebrovascular disease. One recent study examined BPV in older adults during hypocapnia and hypercapnia challenge during perfusion imaging to estimate cerebrovascular reactivity [50], which reflects the ability of the brain’s blood vessels to dilate and constrict in response to vasoactive stimuli and may represent early cerebrovascular dysfunction relevant to cognitive function [29], [42], [60]. In this study, higher BPV was associated with lower cerebrovascular reactivity, even in a sample of community-dwelling older adults with minimal cerebrovascular disease [50].

Cerebrovascular reactivity reflects cerebrovascular function in response to stimuli, but relationships with cerebrovascular function at rest are understudied and could provide information about baseline physiological processes underlying the link between BPV and cerebrovascular disease. Amplitude of low frequency fluctuations (ALFF) is an fMRI-based measure of oscillations of the blood-oxygen-level-dependent (BOLD) signal and is thought to reflect regional spontaneous vascular and neuronal brain activity [4], [64], [75]. In one study, investigators computed vascular density using susceptibility-weighted-imaging for each voxel and, in a voxel-wise analysis, found a correlation of 0.99 with Z-ALFF across subjects [64]. Consistent with this, a recent study found strong spatial correspondence across the brain in blood volume (measured via quantitative PET imaging) and ALFF [9]. A growing number of studies suggest reductions in ALFF, especially in regions vulnerable to Alzheimer’s disease and microvascular insult, are related to cerebrovascular disease burden (e.g., white matter hyperintensities) and cognitive impairment [37], [65], [71]. Additionally, ALFF may decline with age, representing less efficient neurovascular unit function at rest [16]. However, little is known about relationships between BPV and ALFF. Moreover, the medial temporal lobes were recently shown to be vulnerable to elevated BPV and associated lower cerebral perfusion in older adults but not younger adults [55], but relationships with ALFF in these regions are unclear. It is also unknown how BPV may be related to ALFF in other brain regions critical for autonomic nervous system control and early sites of Alzheimer’s disease pathology, such as the insular cortex, amygdala, and anterior cingulate cortex [20]. Findings could help elucidate potential mechanisms underlying the strong link between BPV, cerebrovascular disease, and dementia risk. To investigate these possibilities, we examined the relationship between BPV and concurrent regional ALFF in a sample of community-dwelling older adults and healthy younger adults.

## 2 Methods

### 2.1 Participants

Study data were from the baseline neuroimaging sessions from participants enrolled in a clinical trial (Heart Rate Variability and Emotion Regulation or “HRV-ER” NCT03458910 at ClinicalTrials.gov) at the Emotion and Cognition Lab at the University of Southern California (USC). Participants were recruited via USC’s Healthy Minds and undergraduate subject pools, USC’s online bulletin board, social media, direct mail and flyers. Participants with medical, neurological, or psychiatric conditions were excluded from the study; however, participants taking antidepressant or antianxiety medication were not excluded unless they anticipated a change in treatment during the study. As previously described [70], older adult participants who scored < 16 on the Telephone Screening Protocol (TELE) [12] (cognitive test used to screen for possible dementia) were excluded from the study. The study was approved by the Institutional Review Board at USC and all participants provided their written informed consent.

Of the 193 participants (*n* = 72 older adults [aged 55–80], *n* = 121 younger adults [aged 18–31]) enrolled in the clinical trial, 93 participants (*n* = 44 older adults, *n* = 49 younger adults) had valid BPV and ALFF data from the baseline neuroimaging session. Based on a power analysis for detecting moderate-to-large effect sizes using G*Power [10], multiple linear regression (α = 0.05, 2 covariates) with a sample size of 42 participants will yield 95% power. Therefore, the present investigation is adequately powered to detect moderate-to-large effect sizes in both the older adult and younger adult groups.

### 2.2 Measures

#### 2.2.1 ALFF assessment

Participants underwent brain MRI at the USC’s Dana and David Dornsife Cognitive Neuroimaging Center using a 3 T Siemens® MAGNETOM Prisma MRI scanner and 32-channel head coil, as previously described [30]. T1-weighted magnetization prepared rapid gradient-echo (MP-RAGE) was acquired for high resolution anatomical images (TR = 2300 ms; TE = 2.26 ms; slice thickness = 1.0 mm; flip angle = 9°; field of view = 256 mm, voxel size = 1.0 mm isotropic). Resting state functional MRI (rsfMRI) data were acquired using multi-echo-planar imaging sequence with TR = 2400 mm; TE = 18/35/53 ms; slice thickness = 3.0 mm; flip angle = 75°, FOV = 240 mm; voxel size = 3.0 × 3.0 × 3.0 mm. We acquired 175 volumes (7 min). Participants were instructed to rest, breathe normally, and look at the central white cross on the screen.

To minimize the effects of motion, we employed multi-echo sequences during our rsfMRI scans. Previous work indicates that BOLD T2* signal is linearly dependent on echo time, whereas non-BOLD signal is not echo-time dependent [23]. We applied a multi-echo independent component analysis (ME-ICA) denoising step to distinguish BOLD fluctuations from non-BOLD artifacts including motion and physiology [22], as previously described [30].

After preprocessing by ME-ICA, we performed additional preprocessing steps using FSL, Analysis of Functional NeuroImaging (AFNI), and custom code written in MATLAB [5]. The additional preprocessing steps consisted of: (1) temporal despiking; (2) linear detrending; (3) spatial smoothing (full width at half maximum [FWHM] = 6 mm) and (4) global intensity normalization. We converted the preprocessed images into ASCII files for processing by custom MATLAB code. To remove very low frequencies in the BOLD signal, we applied smoothness priors detrending with parameter lambda = 50, which corresponds to the cutoff frequency 0.01 Hz [62]. We estimated voxel-wise power spectral density (PSD) using the autoregressive (AR) Burg method for each individual scan to capitalize on the improved accuracy of autoregressive approaches relative to the conventional fast Fourier approach [19]. To determine the model order, we first obtained estimates of the best model order using SPSS forecasting ARIMA’s Bayesian information criterion (BIC), autocorrelation function (ACF) and partial-autocorrelation function (PACF) for each participant. Once the best model order for each individual was determined, the modal score across participants (model order = 3) was selected. After voxel-wise PSD estimation, the individual output files were converted into nifti files and the PSD map images were normalized to the MNI152 2-mm template using the transformation matrix from the individual preprocessed images.

ALFF analysis was performed on the obtained power spectrum using the AR method. Since the power at a given frequency is proportional to the square of the amplitude at that frequency component, we calculated the square root of power at each frequency and obtained the sum of the square root of power within the 0.01 ∼ 0.1 Hz frequency range at each voxel to make individual ALFF spatial maps. Z-ALFF is a normalized ALFF relative to the mean amplitude of low frequency fluctuations across voxels. For Z-ALFF spatial maps, the ALFF value at each voxel was transformed to Z score (i.e., minus the global mean value and then divided by the standard deviation [SD]) [75], [76].

**Fig. 1:**
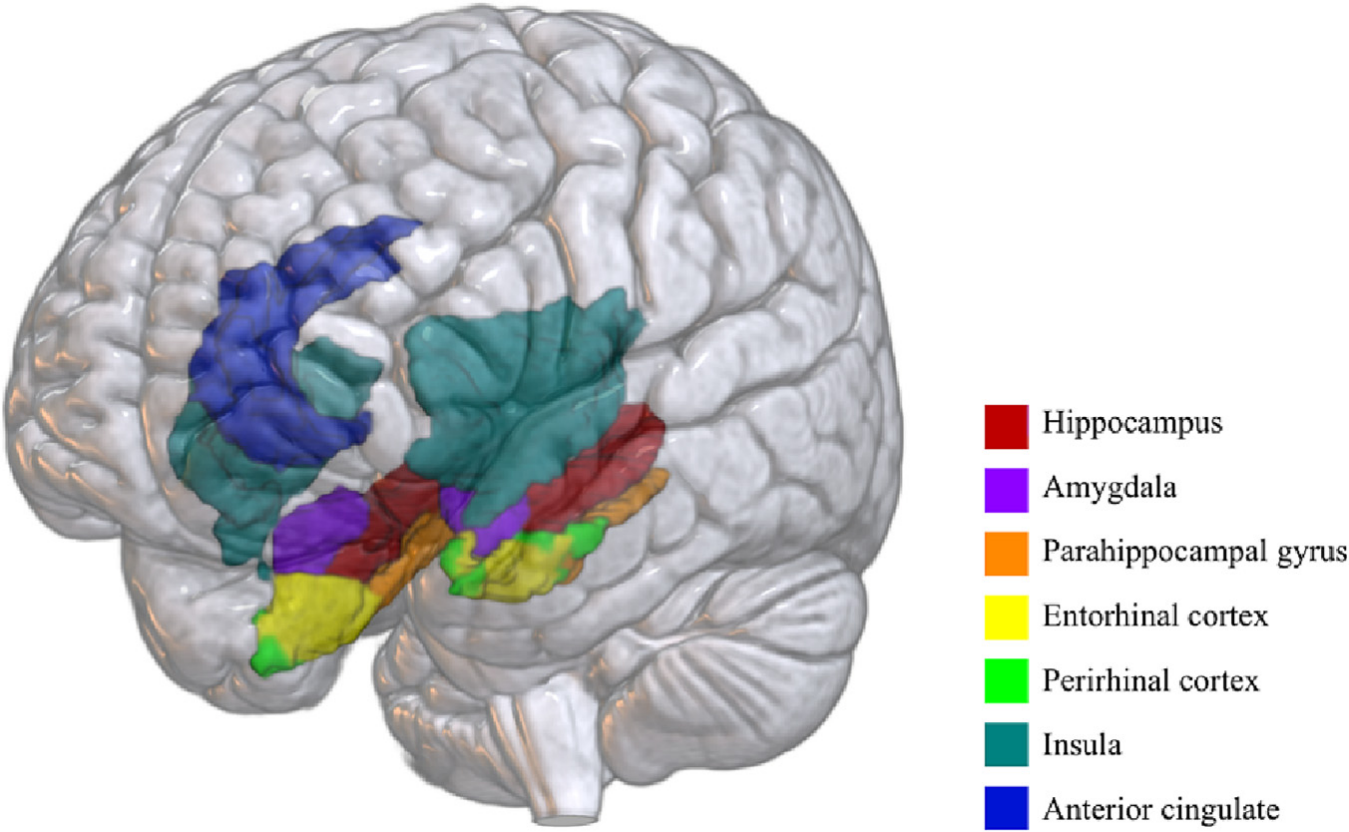
**ROIs** An example of individually segmented ROIs in a younger adult participant. ROIs were defined using automatic segmentation by FreeSurfer ver.6 on a T1-weighted MRI. Abbreviations: ROI = region-of-interest.

We then determined right and left hemispheric Z-ALFF for several *a priori* regions-of-interest (ROI) in the medial temporal lobe recently implicated in studies of BPV and regional cerebral perfusion [54], [55], tau accumulation [51], and gray matter atrophy [52]: hippocampus, parahippocampal gyrus, entorhinal cortex, and perirhinal cortex. We also determined Z-ALFF for brain regions involved in autonomic nervous system regulation relevant to BPV and vulnerable to early stage Alzheimer’s disease pathology [20]: insular cortex, anterior cingulate cortex, and amygdala. Some studies suggest not only laterality but also anterior-posterior dependent effects of the insula on cardiovascular activity [21], [38], [72]. Therefore, we also determined Z-ALFF for anterior vs posterior insula for exploratory analyses. ROI masks were each anatomically defined using that participant’s T1 image (Fig. 1). The segmentation of the right and left amygdala was performed using the FreeSurfer software package version 6 (https://surfer.nmr.mgh.harvard.edu) [11]. Labels from the specific structures were saved as two distinct binary masks in the native space. All files were visually inspected for segmentation accuracy. We used FSL FLIRT to linearly align each participant’s ROI mask to the standard MNI 2-mm brain. We additionally determined Z-ALFF across the right/left whole brain. Whole brain and regional Z-ALFF were then further subdivided into the following frequency bands: slow2 (0.198 - 0.25 Hz), slow3 (0.073 - 0.198 Hz), slow4 (0.027 - 0.073), slow5 (0.01 - 0.027 Hz).

To examine the reproducibility of ALFF within participants, we used bivariate correlation to compare participants’ ALFF from the pre-intervention phase of the study (the focus of the present investigation) vs the post-intervention phase of the study [35]. 79 of the 93 participants had post-intervention ALFF data available. Correlations between intraindividual pre- and post-intervention ALFF (standard 0.01 ∼ 0.1 Hz frequency range) were *r* = 0.531 - 0.755, *p* <.01 in ROIs (Supplementary Table 1).

#### 2.2.2 BP assessment

Brachial artery BP was collected continuously from the left arm using a Biopac® MRI-compatible BP monitoring device during the 7-minute rsfMRI scan. As previously described [55], [50], [58], data were processed offline using a custom pipeline scripted in AcqKnowledge®. Intraindividual BPV was calculated as variation independent of mean (VIM), an index of BPV that is not significantly correlated with mean BP levels [7], [44], [45], [55], [54], [53], [68]. In our sample, we confirmed BPV was not significantly correlated with mean BP levels (bivariate correlation: *r* = -0.04, *p* =.72). VIM was calculated as: VIM = SD/mean*^x^*, where the power *×* was derived from non-linear curve fitting of BP SD against mean BP using the nls package in R Project, as previously described [44], [55], [54], [48], [53]. We focused our investigation on systolic BPV, given recent findings that systolic, and not diastolic, short-term BPV is related to concurrent cerebral blood flow in older adults [55]. However, analyses with diastolic BPV are presented in the Supplementary Materials (Supplementary Table 2). We also calculated mean BP over the scan. Validation of BPV data was not performed. However, our BP collection and BPV calculation methods are consistent with a recent study on BPV and cerebral perfusion [55] that used the same BP monitoring device and MRI scanner and similar study sample. Additionally, the BP monitoring device used has been validated with ultra-sensitive intra-arterial BP monitoring [3], [13], [24].

#### 2.2.3 Heart rate assessment

As previously described [35], [36], [49], [70], participants wore an ear sensor to measure their pulse during the 7-minute rsfMRI scan. Heart rate and heart rate variability (root mean square of successive differences between normal heartbeats [RMSSD]) were calculated using Kubios HRV Premium 3.1 software [61].

#### 2.2.4 Other measurements

The following were determined from a questionnaire at study baseline: antihypertensive medication use (all classes; yes vs no), statin use (yes vs no), diabetes medication use (yes vs no), antidepressant/anti-anxiety medication use (yes vs no), history of smoking (yes vs no), history of alcohol use (yes vs no), history of psychiatric disorder (anxiety, depression; yes vs no), caffeine consumption on the day of the evaluation (yes vs no), stress level on the day of the evaluation vs usual stress level (1–9; 1 = much lower; 5 = same as usual; 9 = much higher), depressive symptoms (Center for Epidemiological Studies – Depression [CESD] [41] total score), anxiety symptoms (State-Trait Anxiety Inventory [STAI] [56]; state-level anxiety total score, trait-level anxiety score).

#### 2.2.5 Data availability statement

Study data are available in the public repository, OpenNeuro, “HRV-ER” (https://openneuro.org/datasets/ds003823).

## 3 Statistical analysis

Multiple linear regression was used to examine the relationship between BPV and regional Z-ALFF (all frequency ranges). Specifically, BPV was entered as the independent variable and Z-ALFF was entered as the dependent variable. BPV elevation has been hypothesized to reflect sympathetic nervous system overactivation [15], [18], [28], [39]. Some studies of older adults suggest sympathetic nervous system functions are largely lateralized to the brain’s right hemisphere [14], [57]. Therefore, we examined relationships between BPV and regional Z-ALFF in the right and left hemisphere separately. Main analyses were stratified by age group (older vs younger). We also tested for potential age differences in a combined model of older and younger adults by interactions with 1) age (years) and 2) age group (older vs younger) (Supplementary Table 3). Additionally, we examined associations between mean BP and regional Z-ALFF in order to directly compare potential effects with BPV (Supplementary Tables 4 and 5). Sensitivity analyses included the following additional covariates: 1) antihypertensive medication use (in models of older adults); 2) statin use (in models of older adults); 3) diabetes medication use (in models of older adults); 4) antidepressant/anti-anxiety medication use; 5) history of smoking; 6) history of alcohol use; 7) caffeine consumption on the day of the evaluation; 8) stress level on the day of the evaluation; 9) mean BP; 10) heart rate; 11) heart rate variability (RMSSD) (Supplementary Table 6). Exploratory analyses examined the relationship between BPV and anterior vs posterior insula Z-ALFF in each hemisphere (Supplementary Table 7). All models controlled for age and sex. All analyses were 2-sided with significance set at *p* <.05. Multiple comparison corrections using the FDR method [2] was set at *p* <.05. All analyses were carried out in R [40].

## 4 Results

Table 1 summarizes clinical and demographic information. Briefly, for the older adult sample, the mean (SD) age was 65.1 (6.6) years, 63.6% were female, and 59.1% were White. For the younger adult sample, the mean (SD) age was 23.3 (3.0), 53.1% were female, and 20.4% were White.

**Table 1:** Demographic information.

	**Older adults (*n* = 44)**	**Younger adults (*n* = 49)**
Age (years)	65.1 (6.6)	23.3 (3.0)
Sex (M/F)	16/28	23/26

Race (*n*, %)		
Asian	8 (18.2%)	31 (63.3%)
Black	8 (18.2%)	3 (6.1%)
White	26 (59.1%)	10 (20.4%)
Other	1 (2.3%)	3 (6.1%)
Bi-/Multiracial	1 (2.3%)	2 (4.1%)

Ethnicity (*n*, %)		
Hispanic	1 (2.3%)	4 (8.2%)
Non-Hispanic	43 (97.7%)	45 (91.8%)
Education (years)	16.6 (2.1)	16.5 (2.3)

Medical history (*n*, %)		
Smoke	11 (25.0%)	3 (6.1%)
Alcohol	19 (43.2%)	20 (40.8%)
Anxiety	1 (2.3%)	3 (6.1%)
Depression	5 (11.4%)	1 (2.0%)

Medication use (*n*, %)		
Antihypertensive	19 (43.2%)	0 (0.0%)
ACE inhibitor	5 (11.4%)	0 (0.0%)
ARB	5 (11.4%)	0 (0.0%)
Calcium-channel blocker	6 (13.6%)	0 (0.0%)
Unknown	4 (9.1%)	0 (0.0%)
Statin	10 (22.7%)	0 (0.0%)
Diabetes	3 (6.8%)	0 (0.0%)
Antidepressant/anti-anxiety	5 (11.4%)	2 (4.1%)
Caffeine† (*n*, %)	26 (59.1%)	15 (30.6%)
Stress*	4.6 (1.5)	5.4 (2.0)
CESD total score	9.7 (8.2)	16.1 (9.6)

STAI total score		
State anxiety	31.6 (10.0)	38.6 (10.2)
Trait anxiety	34.6 (11.9)	43.1 (10.6)

Systolic BP		
Mean	131.8 (24.3)	117.0 (25.7)
BPV	4.3 (1.7)	3.2 (1.7)

Diastolic BP		
Mean	78.0 (21.9)	63.3 (18.2)
BPV	15.4 (2.9)	13.8 (3.4)

Mean (SD) reported unless otherwise indicated.

†caffeine consumption on day of the evaluation.

*stress level on day of the evaluation (1–9; 1 = much lower; 5 = same as usual; 9 = much higher).

Abbreviations: BP = blood pressure; BPV = blood pressure variability; CESD = Center for Epidemiological Studies – Depression; STAI = State-Trait Anxiety Inventory; ACE = angiotensin-converting-enzyme; ARB = angiotensin receptor blocker.

### 4.1 BPV

#### 4.1.1 Older adults

**Fig. 2:**
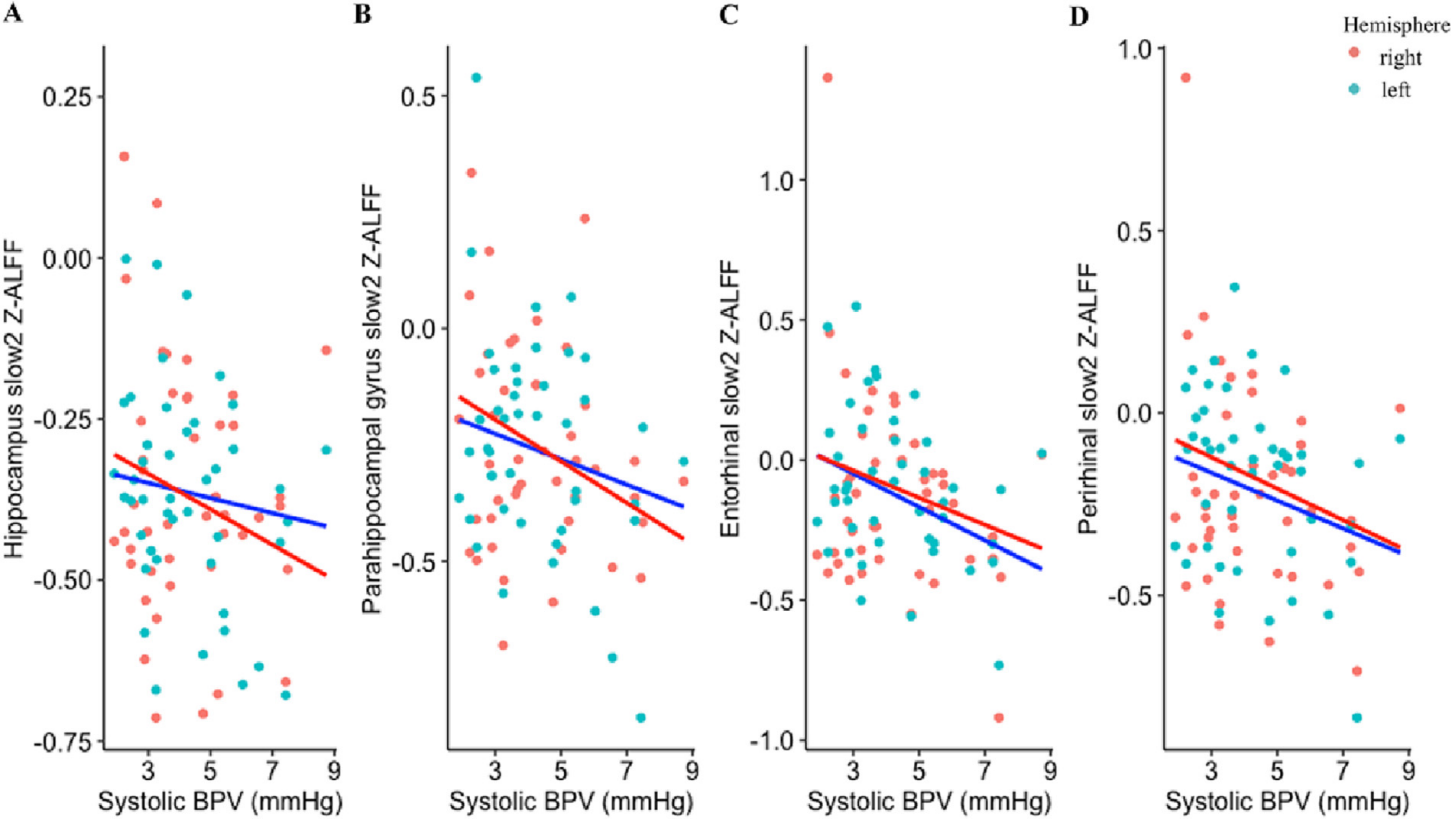
**Elevated systolic BPV is associated with lower concurrent Z-ALFF in all medial temporal regions in older adults.** Scatterplots display the relationship between systolic BPV and slow2 Z-ALFF in **A)** hippocampus **B)** parahippocampal gyrus **C)** entorhinal cortex and **D)** perirhinal cortex in older adults. Right hemisphere values of Z-ALFF are in red; left hemisphere values of Z-ALFF are in blue. Abbreviations: BPV = blood pressure variability. (For interpretation of the references to colour in this figure legend, the reader is referred to the web version of this article.)

As reported in Table 2 and shown in Fig. 2, elevated systolic BPV in older adults was associated with significantly lower Z-ALFF in all medial temporal regions (i.e., hippocampus, parahippocampal gyrus, entorhinal cortex, perirhinal cortex). Associations were more robust in the right hemisphere relative to the left hemisphere, and in the faster frequency ranges (i.e., slow2, slow3) when compared to the slower frequency ranges (i.e., slow4, slow5). Higher systolic BPV in older adults was also associated with lower slow5 Z-ALFF in the right anterior cingulate cortex (data not shown). There were no significant associations between systolic BPV in older adults and Z-ALFF in the right/left whole brain, amygdala, or insular cortex. Findings with diastolic BPV were generally similar, with additional associations observed in right whole brain and left perirhinal cortex (Supplementary Table 2). Diastolic BPV was not associated with Z-ALFF in the anterior cingulate cortex (Supplementary Table 2).

**Table 2:** Model estimates of BPV predicting regional Z-ALFF in older adults.

	**ALFF (0.01 – 0.10 Hz)**	**Slow2 (0.198 - 0.25 Hz)**	**Slow3 (0.073 - 0.198 Hz)**	**Slow4 (0.027 - 0.073 Hz)**	**Slow5 (0.01 - 0.027 Hz)**
**Region**					
L WB	0.03 [-0.28, 0.34]	0.08 [-0.19, 0.35]	0.10 [-0.21, 0.41]	0.03 [-0.29, 0.34]	-0.01 [-0.31, 0.29]
R WB	-0.25 [-0.51, 0.01]	-0.28 [-0.67, 0.01]	-0.24 [-0.51, 0.02]	-0.25 [-0.51, 0.01]	-0.22 [-0.49, 0.06]
L HC	-0.11 [-0.41, 0.19]	-0.12 [-0.42, 0.18]	-0.12 [-0.43, 0.19]	-0.11 [-0.42, 0.19]	-0.004 [-0.30, 0.29]
R HC	**-0.38 [-0.65, -0.11]**	**-0.29 [-0.54, -0.03]**	**-0.34 [-0.63, -0.05]**	**-0.37 [-0.65, -0.09]**	-0.27 [-0.56, 0.03]
L PHG	-0.13 [-0.41, 0.15]	-0.21 [-0.50, 0.08]	-0.18 [-0.45, 0.11]	-0.13 [-0.41, 0.15]	-0.06 [-0.34, 0.21]
R PHG	**-0.33 [-0.64, -0.02]**	**-0.34 [-0.63, -0.04]**	-0.30 [-0.64, 0.04]	**-0.33 [-0.64, -0.03]**	-0.25 [-0.56, 0.06]
L EC	-0.28 [-0.60, 0.04]	**-0.32 [-0.65, -0.002]**	-0.25 [-0.57, 0.07]	-0.32 [-0.64, 0.002]	-0.30 [-0.62, 0.02]
R EC	-0.27 [-0.57, 0.03]	**-0.29 [-0.56, -0.02]**	-0.36 [-0.64, -0.09]	-0.25 [-0.56, 0.06]	-0.19 [-0.50, 0.40]
L PC	-0.21 [-0.51, 0.09]	-0.26 [-0.58, 0.06]	-0.20 [-0.50, 0.09]	-0.25 [-0.55, 0.05]	-0.21 [-0.51, 0.08]
R PC	-0.24 [-0.51, 0.03]	**-0.29 [-0.56, -0.03]**	**-0.34 [-0.62, -0.05]**	-0.23 [-0.49, 0.04]	-0.19 [-0.46, 0.09]
L AM	-0.21 [-0.52, 0.11]	-0.20 [-0.48, 0.08]	-0.18 [-0.49, 0.13]	-0.21 [-0.52, 0.10]	-0.19 [-0.51, 0.12]
R AM	-0.17 [-0.48, 0.14]	-0.17 [-0.44, 0.11]	-0.19 [-0.51, 0.13]	-0.18 [-0.48, 0.13]	-0.16 [-0.48, 0.16]
L IN	0.03 [-0.28, 0.34]	-0.12 [-0.40, 0.17]	-0.04 [-0.35, 0.28]	0.03 [-0.29, 0.34]	-0.02 [-0.33, 0.29]
R IN	-0.17 [-0.49, 0.14]	-0.07 [-0.39, 0.25]	-0.09 [-0.42, 0.24]	-0.18 [-0.50, 0.13]	-0.18 [-0.49, 0.14]
L ACC	-0.11 [-0.39, 0.17]	-0.04 [-0.37, 0.30]	-0.09 [-0.40, 0.23]	-0.11 [-0.40, 0.17]	-0.16 [-0.41, 0.10]
R ACC	-0.20 [-0.47, 0.07]	-0.06 [-0.36, 0.24]	-0.09 [-0.38, 0.21]	-0.23 [-0.50, 0.05]	**-0.26 [-0.51, -0.01]**

Standardized beta (ß) and 95% confidence intervals shown unless otherwise indicated.

Bolded items indicate BPV is significantly associated with regional Z-ALFF.

Models covaried for age and sex.

Abbreviations: L = left hemisphere: R = right hemisphere; WB = whole brain; HC = hippocampus; PHG = parahippocampal gyrus; EC = entorhinal cortex; PC = perirhinal cortex; AM = amygdala; IN = insular cortex; ACC = anterior cingulate cortex; ALFF = amplitude of low frequency fluctuations.

#### 4.1.2 Younger adults

**Fig. 3:**
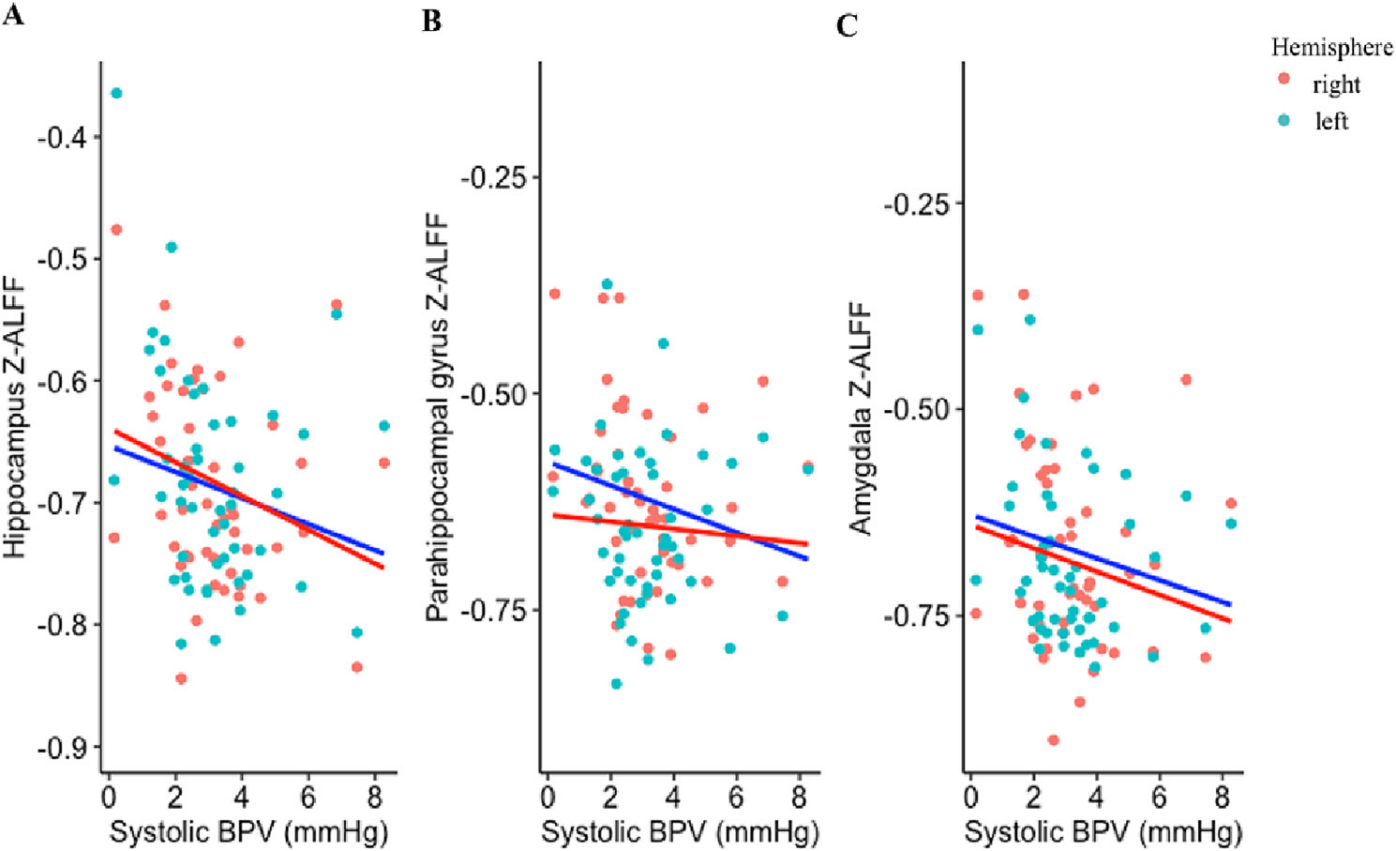
**Elevated systolic BPV is associated with lower concurrent Z-ALFF in hippocampus, parahippocampal gyrus, and amygdala in younger adults** Scatterplots display the relationship between systolic BPV and Z-ALFF in **A)** hippocampus **B)** parahippocampal gyrus and **C)** amygdala in younger adults. Right hemisphere values of Z-ALFF are in red; left hemisphere values of Z-ALFF are in blue. Abbreviations: BPV = blood pressure variability. (For interpretation of the references to colour in this figure legend, the reader is referred to the web version of this article.)

As summarized in Table 3 and shown in Fig. 3, elevated systolic BPV in younger adults was related to significantly lower Z-ALFF in hippocampus, parahippocampal gyrus, and amygdala. Findings were present in both hemispheres and mostly in the slow3 and slow4 frequency ranges (vs slow2 and slow5). Systolic BPV in younger adults was not significantly associated with Z-ALFF in right/left whole brain, entorhinal cortex, perirhinal cortex, insular cortex, or anterior cingulate cortex. Findings with diastolic BPV were consistent, with additional associations observed in left perirhinal cortex and right insular cortex in some frequency ranges (Supplementary Table 2).

**Table 3:** Model estimates of BPV predicting regional Z-ALFF in younger adults.

	**ALFF (0.01 – 0.10 Hz)**	**Slow2 (0.198 - 0.25 Hz)**	**Slow3 (0.073 - 0.198 Hz)**	**Slow4 (0.027 - 0.073 Hz)**	**Slow5 (0.01 - 0.027 Hz)**
**Region**					
L WB	-0.09 [-0.38, 0.21]	0.08 [-0.20, 0.36]	0.03 [-0.26, 0.31]	-0.11 [-0.40, 0.18]	-0.08 [-0.39, 0.22]
R WB	-0.01 [-0.29, 0.27]	0.14 [-0.14, 0.42]	0.04 [-0.23, 0.31]	-0.02 [-0.30, 0.26]	0.02 [-0.26, 0.29]
L HC	**-0.28 [-0.54, -0.01]**	-0.19 [-0.46, 0.07]	**-0.27 [-0.53, -0.01]**	**-0.28 [-0.54, -0.02]**	-0.20 [-0.47, 0.07]
R HC	**-0.31 [-0.61, -0.01]**	-0.24 [-0.50, 0.02]	**-0.30 [-0.58, -0.03]**	**-0.31 [-0.62, -0.003]**	-0.21 [-0.52, 0.10]
L PHG	**-0.24 [-0.47, -0.01]**	-0.15 [-0.42, 0.13]	-0.22 [-0.46, 0.03]	**-0.24 [-0.47, -0.01]**	-0.19 [-0.43, 0.04]
R PHG	-0.12 [-0.41, 0.16]	-0.13 [-0.40, 0.13]	-0.16 [-0.45, 0.14]	-0.12 [-0.40, 0.16]	-0.06 [-0.34, 0.21]
L EC	-0.15 [-0.46, 0.16]	-0.09 [-0.37, 0.19]	-0.13 [-0.42, 0.16]	-0.16 [-0.47, 0.15]	-0.10 [-0.40, 0.20]
R EC	-0.16 [-0.45, 0.12]	-0.06 [-0.30, 0.19]	-0.11 [-0.38, 0.16]	-0.18 [-0.47, 0.11]	-0.17 [-0.47, 0.13]
L PC	-0.22 [-0.50, 0.07]	-0.20 [-0.48, 0.07]	-0.24 [-0.52, 0.03]	-0.21 [-0.50, 0.08]	-0.15 [-0.44, 0.15]
R PC	-0.14 [-0.44, 0.16]	-0.17 [-0.42, 0.07]	-0.17 [-0.45, 0.11]	-0.14 [-0.44, 0.16]	-0.14 [-0.43, 0.16]
L AM	-0.28 [-0.57, 0.02]	-0.19 [-0.46, 0.08]	**-0.30 [-0.55, -0.05]**	-0.27 [-0.57, 0.03]	-0.17 [-0.48, 0.13]
R AM	**-0.27 [-0.53, -0.01]**	**-0.31 [-0.55, -0.06]**	**-0.33 [-0.58, -0.09]**	-0.26 [-0.53, 0.01]	-0.14 [-0.40, 0.12]
L IN	-0.09 [-0.33, 0.14]	-0.19 [-0.46, 0.09]	-0.17 [-0.41, 0.07]	-0.09 [-0.32, 0.15]	-0.09 [-0.32, 0.15]
R IN	-0.27 [-0.54, 0.01]	-0.18 [-0.42, 0.06]	-0.22 [-0.46, 0.02]	-0.27 [-0.55, 0.02]	-0.24 [-0.53, 0.05]
L ACC	-0.01 [-0.28, 0.26]	-0.03 [-0.32, 0.26]	0.003 [-0.28, 0.29]	-0.02 [-0.28, 0.25]	-0.02 [-0.31, 0.26]
R ACC	-0.01 [-0.35, 0.33]	-0.11 [-0.45, 0.23]	-0.04 [-0.37, 0.29]	-0.02 [-0.36, 0.32]	0.03 [-0.30, 0.35]

Standardized beta (ß) and 95% confidence intervals shown unless otherwise indicated.

Bolded items indicate BPV is significantly associated with regional Z-ALFF.

Models covaried for age and sex.

Abbreviations: L = left hemisphere: R = right hemisphere; WB = whole brain; HC = hippocampus; PHG = parahippocampal gyrus; EC = entorhinal cortex; PC = perirhinal cortex; AM = amygdala; IN = insular cortex; ACC = anterior cingulate cortex; ALFF = amplitude of low frequency fluctuations.

#### 4.1.3 Age & age group interactions

There were significant systolic BPV × age group interactions on slow3 Z-ALFF in the right entorhinal cortex and slow2 Z-ALFF in the right whole brain, where higher systolic BPV in older adults was associated with lower Z-ALFF. In all other models that combined older and young adults, there were no significant systolic BPV × age or systolic BPV × age group interactions on Z-ALFF (Supplementary Table 3).

### 4.2 Mean BP

Mean systolic BP was not significantly associated with Z-ALFF in any region in older adults or younger adults (Supplementary Tables 4 and 5).

### 4.3 Sensitivity analyses

Systolic BPV findings in older adults remained largely unchanged in sensitivity analyses additionally controlling for 1) antihypertensive medication use; 2) diabetes medication use; 3) antidepressant/anti-anxiety medication use; 4) history of smoking; 5) history of alcohol use; 6) caffeine consumption on the day of the evaluation; 7) stress level on the day of the evaluation; 8) mean BP; 9) heart rate; and 10) heart rate variability (RMSSD) (Supplementary Table 6). Findings in older adults were attenuated or no longer significant when controlling for statin use (Supplementary Table 6). Associations in younger adults were also largely unchanged in sensitivity analyses, but findings were attenuated or no longer significant when additionally controlling for history of smoking, heart rate, and heart rate variability (RMSSD) (Supplementary Table 6). Main systolic BPV findings in older and younger adults were no longer significant after FDR correction.

### 4.4 Exploratory analyses

Higher systolic BPV was related to lower Z-ALFF in the right posterior insula in younger adults, but all other anterior vs posterior insula exploratory analyses were not significant (Supplementary Table 7).

## 5 Discussion

Study findings suggest higher BPV, independent of mean BP, is associated with lower Z-ALFF, especially in the medial temporal lobe and in older adults. Several studies indicate BPV elevation is associated with frank cerebrovascular disease burden observable on MRI and postmortem evaluation [26], [48], [63], and the present results add to this literature by suggesting BPV may also be related to early cerebrovascular dysfunction in regions highly vulnerable to Alzheimer’s disease.

Arterial stiffness is one mechanism thought to underly relationships between BPV, cerebrovascular disease, and dementia risk. As arteries stiffen, their ability to dampen pulse wave dynamics tends to decrease, which could allow BP levels to fluctuate more widely [18], [74]. Chronic large oscillations in BP levels could have a “tsunami effect” [46] on vessel walls, promoting arterial remodeling, changes in the blood–brain barrier, and resulting disruptions in neurovascular functioning, which could be related to the present findings of reduced spontaneous brain activity. Therefore, it is possible that higher BPV may indirectly alter neurovascular coupling as captured by MRI measures such as lower ALFF.

Reductions in ALFF were most pronounced in medial temporal lobes, which support memory function and are known to be highly sensitive to disruptions in cerebral blood flow and an early site for neurofibrillary changes in Alzheimer’s disease [6], [47]. Interestingly, the present findings showing a relationship between higher BPV and reduced ALFF are strikingly similar to recent findings between higher BPV and reduced cerebral blood flow in these same regions in older adults [55]. Moreover, other BPV studies in older adults implicate gray matter atrophy [27], [52], tau accumulation [25], [48], and cerebral perfusion decline in the medial temporal lobes [54]. Higher BPV in younger adults was also related to lower ALFF in the amygdala and insular cortex, regions involved in control of the autonomic nervous system [20], [33]. Together these findings suggest BPV may be an understudied vascular factor related to both vascular and neuronal brain changes in aging, especially in highly vulnerable regions critical for cognitive and autonomic function. Alternatively, it is possible that neurodegenerative effects on autonomic control centers such as the amygdala and insular cortex may drive fluctuations in BP [20], [34], [33], and changes in ALFF. The present study is both cross-sectional and observational and future work with longitudinal and/or interventional designs will help clarify the role of BPV in brain health.

Associations between BPV and ALFF in older adults were more robust in the right hemisphere, while younger adults showed associations similarly across hemispheres, albeit in fewer brain regions. BPV elevation has been hypothesized to reflect overactivation of the sympathetic nervous system [28], which in some studies has been lateralized to the right hemisphere [14], [57]. Our findings support this hypothesis, at least in older adults. This could suggest that these regions are linked with sympathetic nervous system overactivation, which is relevant to stroke risk [73]. However, more research is warranted. Nevertheless, findings may be relevant to therapeutic intervention. For example, some studies indicate that different classes of antihypertensive agents have differential effects on BPV and risk of stroke, independent of mean BP levels [43], [66]. Although the present study was not adequately powered to test this possibility as it relates to ALFF, this remains an important area for future research. Additionally, mean BP, which is a more traditionally studied index of BP and often the target in intervention studies [67], was not significantly associated with ALFF in any region in older adults or younger adults. This highlights the specific contribution of BPV, and not mean BP, to ALFF and provides new information on the relationship between BPV and cerebrovascular dysfunction and disease. There were almost no significant BPV × age or BPV × age group interactions on ALFF in models that combined older and younger adults. Additionally, stratified analyses suggested that higher BPV was associated with lower ALFF in both age groups, albeit less robustly and in fewer subregions in younger adults. This suggests BPV may be related to cerebrovascular function in both older and younger adults, but that the impact of large BP fluctuations on brain health may be greater in older adults.

The study used a novel approach of collecting BP continuously during rsfMRI, which allowed us to examine the relationship between BPV and concurrent spontaneous vascular and neuronal brain activity. Prior work has shown that BPV elevation is associated with cerebrovascular lesions on structural MRI [26], [63], and one recent study suggests higher BPV is related to reduced cerebrovascular reactivity during hypocapnia and hypercapnia challenge during perfusion MRI [50]. The present study adds to this work by using functional MRI to delineate relationships with early markers of cerebrovascular dysfunction at rest. We studied older and younger adults to examine potential age-related differences. The study is also strengthened by the racial/ethnic diversity of the study sample. Additionally, we were able to characterize – and control for in sensitivity analyses – various factors relevant to autonomic function, including caffeine intake and stress levels on the day of the evaluation. There are several limitations worth noting. First, the study sample was relatively small. Relatedly, we were not able to examine relationships with Alzheimer’s disease risk gene apolipoprotein e4. Several recent studies indicate BPV may be associated with important markers of Alzheimer’s disease, especially in apolipoprotein e4 carriers [52], [51], increasingly appreciated to have vulnerability to vascular factors [1], [31]. Studies with larger samples will be better able to investigate this possibility as it relates to ALFF. Compared to functional connectivity, ALFF is a relatively less well-studied marker of cerebrovascular function captured by rsfMRI. However, ALFF reflects fluctuations in the BOLD signal itself, potentially revealing properties more relevant to the variability in neurovascular unit function vs the strength of connections between neurons driven by functional hyperemia. Similar to the study of BPV independent of mean BP levels, ALFF may offer insights into function beyond mean levels of neural activity. Additional limitations include the fact that older adults in the study were living independently in the community and had TELE scores ≥ 16, but other characteristics of cognitive function were not used as inclusion/exclusion criteria. The TELE is a brief telephone-based test that is used to screen for possible dementia but it does not rule out the possibility of mild cognitive impairment. Therefore, some of the older adult participants may have had mild cognitive impairment, which could affect the study findings. However, our older adult sample was comparable to many other BPV studies using community-based samples that may or may not have included individuals with mild cognitive impairment [8], [26], [59]. Nevertheless, BPV may be higher in those with mild cognitive impairment when compared to older adults with normal cognition [53], at least when BPV is measured over longer time intervals. Future work exploring relationships in well-characterized older adult samples has the potential to add to our understanding of BPV as an understudied vascular risk factor for dementia.

## 6 Conclusions

Elevated BPV was associated with lower ALFF especially in medial temporal regions and older adults. Findings add to ongoing work detailing relationships between BPV, cerebrovascular disease, and dementia by exploring associations with early markers of cerebrovascular dysfunction at rest. BPV may be an understudied vascular factor for cerebrovascular changes in aging relevant to cognitive function.

## Declaration of Competing Interest

The authors declare that they have no known competing financial interests or personal relationships that could have appeared to influence the work reported in this paper.
