# Supplementary material for: Short-term blood pressure variability is inversely related to regional amplitude of low frequency fluctuations in older and younger adults
Source: Aging Brain. 2023 Jul 11;4:100085. doi: 10.1016/j.nbas.2023.100085 (PMC10362312; doi:10.1016/j.nbas.2023.100085)
Supplement: Supplementary Data 1 [file mmc1.docx]

SUPPLEMENTARY MATERIALS

**Supplementary Table 1.** – Reproducibility of ALFF and Z-ALFF

**Supplementary Table 2. -** Diastolic BPV predicting regional Z-ALFF

**Supplementary Table 3.** – Systolic BPV x age and systolic BPV x age group interactions predicting regional Z-ALFF.

**Supplementary Table 4.** –Mean systolic BP predicting regional Z-ALFF in older adults

**Supplementary Table 5.** –Mean systolic BP predicting regional Z-ALFF in younger adults

**Supplementary Table 6.** –Systolic BPV predicting regional Z-ALFF in sensitivity analyses controlling for additional covariates

**Supplementary Table 7.** – Systolic BPV predicting Z-ALFF in anterior and posterior insula

**Supplementary Table 1.**

Test-retest reliability of ALFF and Z-ALFF in standard ALFF range (.01 – .10 Hz) in ROIs (N=79).

|  | **ALFF** | **Z-ALFF** |
| --- | --- | --- |
| **Region** |  |  |
| WB | .741** |  |
| HC | .648** | .531** |
| PHG | .638** | .625** |
| EC | .628** | .658** |
| PC | .563** | .563** |
| AM | .601** | .632** |
| IN | .716** | .583** |
| ACC | .755** | .582** |

** Correlation is significant at the 0.01 level (2-tailed).

ALFF and Z-ALFF values averaged across left and right hemisphere.

Abbreviations: WB = whole brain; HC = hippocampus; PHG = parahippocampal gyrus; EC = entorhinal cortex; PC = perirhinal cortex; AM = amygdala; IN = insular cortex; ACC = anterior cingulate cortex; ALFF = amplitude of low frequency fluctuations

**Supplementary Table 2.**

Model estimates of diastolic BPV predicting regional Z-ALFF.

|  | **ALFF**  **(.01 – .10 Hz)** | **Slow2**  **(.198 - .25 Hz)** | **Slow3**  **(.073 - .198 Hz)** | **Slow4**  **(.027 - .073 Hz)** | **Slow5**  **(.01 - .027 Hz)** |
| --- | --- | --- | --- | --- | --- |
|  |  |  |  |  |  |
| **Older adults** |  |  |  |  |  |
| **Region** |  |  |  |  |  |
| L WB | -.03 [-.34, .29] | .01 [-.27, .28] | .01 [-.31, .32] | -.04 [-.35, .28] | -.08 [-.38, .23] |
| R WB | **-.27 [-.53, -.004]** | -.22 [-.52, .07] | -.19 [-.46, .09] | **-.27 [-.55, -.01]** | -.19 [-.47, .08] |
| L HC | -.24 [-.54, .06] | -.17 [-.47, .13] | -.20 [-.50, .11] | -.24 [-.54, .06] | -.08 [-.38, .22] |
| R HC | **-.39 [-.66, -.11]** | **-.27 [-.53, -.01]** | **-.34 [-.63, -.04]** | **-.38 [-.67, -.10]** | -.23 [-.53, .07] |
| L PHG | -.18 [-.45, .10] | -.15 [-.45, .15] | -.07 [-.36, .22] | -.19 [-.47, .09] | -.09 [-.37, .18] |
| R PHG | **-.33 [-.64, -.01]** | -.24 [-.55, .07] | -.22 [-.57, .13] | **-.34 [-.65, -.04]** | -.19 [-.51, .12] |
| L EC | **-.35 [-.66, -.03]** | -.25 [-.58, .08] | -.22 [-.54, .11] | **-.37 [-.69, -.06]** | -.32 [-.64, .002] |
| R EC | -.29 [-.59, .01] | -.22 [-.50, .06] | -.29 [-.58, .001] | -.27 [-.58, .04] | -.20 [-.50, .11] |
| L PC | **-.31 [-.60, -.01]** | -.30 [-.62, .01] | -.26 [-.55, .04] | **-.34 [-.63, -.04]** | -.20 [-.50, .10] |
| R PC | **-.30 [-.56, -.03]** | -.26 [-.54, .01] | **-.31 [-.60, -.02]** | **-.29 [-.56, -.03]** | -.22 [-.50, .05] |
| L AM | -.18 [-.50, .13] | -.18 [-.46, .10] | -.17 [-.49, .14] | -.17 [-.49, .15] | -.11 [-.44, .21] |
| R AM | -.12 [-.44, .19] | -.11 [-.39, .17] | -.11 [-.44, .22] | -.12 [-.43, .19] | -.09 [-.41, .24] |
| L IN | .06 [-.26, .37] | -.13 [-.42, .16] | -.03 [-.35, .28] | .06 [-.25, .38] | .07 [-.25, .38] |
| R IN | -.10 [-.43, .22] | -.05 [-.37, .27] | -.05 [-.39, .28] | -.11 [-.43, .21] | -.07 [-.39, .25] |
| L ACC | -.002 [-.29, .28] | .03 [-.31, .36] | .01 [-.31, .33] | -.002 [-.29, .29] | -.03 [-.29, .23] |
| R ACC | .03 [-.25, .30] | .02 [-.29, .32] | .07 [-.23, .37] | .02 [-.27, .30] | -.04 [-.31, .22] |
| **Younger adults** |  |  |  |  |  |
| **Region** |  |  |  |  |  |
| L WB | .02 [-.28, .32] | .07 [-.22, .37] | .05 [-.26, .35] | .01 [-.29, .30] | .05 [-.27, .36] |
| R WB | -.13 [-.42, .17] | .10 [-.20, .40] | .01 [-.28, .30] | -.14 [-.43, .16] | -.11 [-.40, .18] |
| L HC | **-.36 [-.63, -.09]** | -.24 [-.52, .05] | **-.29 [-.56, -.01]** | **-.38 [-.64, -.11]** | -.25 [-.52, .03] |
| R HC | **-.44 [-.74, -.13]** | **-.29 [-.56, -.02]** | **-.33 [-.62, -.05]** | **-.45 [-.76, -.14]** | **-.32 [-.63, -.01]** |
| L PHG | **-.31 [-.54, -.08]** | -.18 [-.47, .11] | -.23 [-.49, .03] | **-.32 [-.54, -.09]** | **-.28 [-.52, -.04]** |
| R PHG | -.13 [-.43, .18] | -.12 [-.40, .17] | -.12 [-.43, .19] | -.13 [-.42, .17] | -.06 [-.36, .23] |
| L EC | -.25 [-.57, .08] | -.09 [-.38, .19] | -.14 [-.44, .16] | -.26 [-.59, .07] | -.18 [-.50, .15] |
| R EC | -.10 [-.41, .21] | .01 [-.26, .27] | -.01 [-.30, .28] | -.13 [-.44, .18] | -.08 [-.40, .24] |
| L PC | **-.34 [-.64, -.05]** | -.23 [-.52, .06] | -.25 [-.54, .04] | **-.35 [-.65, -.05]** | -.27 [-.58, .04] |
| R PC | -.18 [-.50, .14] | -.11 [-.38, .16] | -.12 [-.42, .19] | -.20 [-.52, .12] | -.19 [-.50, .13] |
| L AM | **-.37 [-.67, -.08]** | -.22 [-.51, .07] | **-.33 [-.59, -.07]** | **-.38 [-.68, -.08]** | -.25 [-.56, .06] |
| R AM | **-.31 [-.59, -.04]** | -.27 [-.54, .003] | -.27 [-.54, .01] | **-.32 [-.60, -.04]** | -.18 [-.45, .10] |
| L IN | -.11 [-.35, .14] | -.20 [-.49, .09] | -.21 [-.46, .04] | -.09 [-.33, .16] | -.08 [-.32, .17] |
| R IN | -.28 [-.56, .01] | -.18 [-.44, .08] | -.19 [-.44, .05] | **-.29 [-.58, -.004]** | -.24 [-.54, .06] |
| L ACC | .03 [-.26, .31] | .04 [-.26, .35] | .05 [-.26, .36] | .02 [-.26, .31] | .002 [-.30, .31] |
| R ACC | .05 [-.31, .41] | -.07 [-.43, .29] | -.003 [-.35, .35] | .05 [-.31, .41] | .08 [-.27, .43] |

Standardized beta (ß) and 95% confidence intervals shown unless otherwise indicated.

Bolded items indicate diastolic BPV is significantly associated with regional Z-ALFF.

Models covaried for age and sex.

Abbreviations: L = left hemisphere: R = right hemisphere; WB = whole brain; HC = hippocampus; PHG = parahippocampal gyrus; EC = entorhinal cortex; PC = perirhinal cortex; AM = amygdala; IN = insular cortex; ACC = anterior cingulate cortex; ALFF = amplitude of low frequency fluctuations

**Supplementary Table 3.**

Model estimates of the interaction of systolic BPV x age and BPV x age group predicting regional Z-ALFF.

|  | **ALFF**  **(.01 – .10 Hz)** | **Slow2**  **(.198 - .25 Hz)** | **Slow3**  **(.073 - .198 Hz)** | **Slow4**  **(.027 - .073 Hz)** | **Slow5**  **(.01 - .027 Hz)** |
| --- | --- | --- | --- | --- | --- |
| **Region** |  |  |  |  |  |
| L WB |  |  |  |  |  |
| BPV x age | .002 [-.01, .01] | -.001 [-.01, .01] | .001 [-.01, .01] | .003 [-.01, .01] | .001 [-.01, .01] |
| BPV x age group | -.10 [-.50, .30] | .05 [-.33, .44] | -.01 [-.41, .39] | -.12 [-.52, .28] | -.07 [-.48, .34] |
| R WB |  |  |  |  |  |
| BPV x age | -.01 [-.02, .004] | -.01 [-.02, .002] | -.01 [-.02, .004] | -.01 [-.02, .01] | -.01 [-.02, .01] |
| BPV x age group | .26 [-.15, .67] | **.47 [.05, .89]** | .36 [-.06, .78] | .24 [-.16, .65] | .23 [-.18, .63] |
| L HC |  |  |  |  |  |
| BPV x age | .002 [-.01, .01] | .002 [-.01, .01] | .003 [-.01, .01] | .002 [-.01, .01] | .003 [-.01, .01] |
| BPV x age | -.14 [-.56, .28] | -.06 [-.47, .36] | -.11 [-.52, .31] | -.14 [-.56, .28] | -.21 [-.63, .21] |
| R HC |  |  |  |  |  |
| BPV x age | -.004 [-.01, .01] | -.003 [-.01, .01] | -.003 [-.01, .01] | -.004 [-.02, .01] | -.003 [-.01, .01] |
| BPV x age group | .04 [-.38, .47] | .04 [-.35, .42] | .05 [-.36, .46] | .03 [-.40, .47] | -.001 [-.44, .44] |
| L PHG |  |  |  |  |  |
| BPV x age | .002 [-.01, .01] | -.001 [-.01, .01] | .001 [-.01, .01] | .002 [-.01, .01] | .003 [-.01, .01] |
| BPV x age group | -.11 [-.48, .26] | .07 [-.35, .48] | -.02 [-.41, .37] | -.12 [-.49, .26] | -.12 [-.49, .25] |
| R PHG |  |  |  |  |  |
| BPV x age | -.01 [-.02, .002] | -.01 [-.02, .002] | -.01 [-.02, .002] | -.01 [-.02, .002] | -.01 [-.02, .004] |
| BPV x age group | .25 [-.17, .66] | .28 [-.14, .70] | .33 [-.15, .81] | .23 [-.17, .63] | .19 [-.22, .60] |
| L EC |  |  |  |  |  |
| BPV x age | -.002 [-.01, .01] | -.01 [-.02, .01] | -.002 [-.01, .01] | -.003 [-.01, .01] | -.003 [-.01, .01] |
| BPV x age group | .20 [-.24, .65] | .31 [-.14, .75] | .21 [-.23, .65] | .23 [-.22, .67] | .26 [-.19, .70] |
| R EC |  |  |  |  |  |
| BPV x age | -.004 [-.01, .004] | -.01 [-.02, .003] | -.01 [-.02, .001] | -.004 [-.01, .01] | -.001 [-.01, .01] |
| BPV x age group | .23 [-.14, .60] | .32 [-.08, .71] | **.38 [.01, .75]** | .19 [-.18, .57] | .11 [-.28, .50] |
| L PC |  |  |  |  |  |
| BPV x age | .001 [-.01, .01] | -.002 [-.01, .01] | .002 [-.01, .01] | .0002 [-.01, .01] | .0003 [-.01, .01] |
| BPV x age group | .06 [-.38, .49] | .14 [-.33, .56] | .04 [-.37, .46] | .09 [-.35, .52] | .11 [-.33, .55] |
| R PC |  |  |  |  |  |
| BPV x age | -.004 [-.01, .01] | -.004 [-.01, .01] | -.007 [-.02, .003] | -.004 [-.01, .01] | -.002 [-.01, .01] |
| BPV x age group | .22 [-.16, .60] | .18 [-.22, .58] | .31 [-.09, .71] | .20 [-.18, .58] | .16 [-.24, .55] |
| L AM |  |  |  |  |  |
| BPV x age | -.01 [-.02, .01] | -.003 [-.01, .01] | -.002 [-.01, .01] | -.01 [-.02, .01] | -.01 [-.02, .01] |
| BPV x age group | .03 [-.43, .49] | .09 [-.34, .51] | .01 [-.43, .45] | .03 [-.43, .49] | .07 [-.39, .53] |
| R AM |  |  |  |  |  |
| BPV x age | -.002 [-.01, .01] | .002 [-.01, .01] | .0002 [-.01, .01] | -.003 [-.01, .01] | -.005 [-.02, .01] |
| BPV x age group | -.03 [-.44, .38] | -.12 [-.50, .27] | -.09 [-.50, .32] | -.02 [-.43, .39] | .06 [-.36, .48] |
| L IN |  |  |  |  |  |
| BPV x age | .001 [-.01, .01] | .001 [-.01, .01] | .001 [-.01, .01] | .001 [-.01, .01] | .0002 [-.01, .01] |
| BPV x age group | -.11 [-.51, .30] | -.05 [-.46, .37] | -.07 [-.49, .36] | -.10 [-.50, .31] | -.06 [-.45, .34] |
| R IN |  |  |  |  |  |
| BPV x age | -.002 [-.01, .01] | .0002 [-.01, .01] | .00005 [-.01, .01] | -.002 [-.01, .01] | -.001 [-.01, .01] |
| BPV x age group | -.08 [-.53, .37] | -.12 [-.51, .28] | -.11 [-.52, .31] | -.07 [-.52, .39] | -.08 [-.53, .37] |
| L ACC |  |  |  |  |  |
| BPV x age | -.004 [-.01, .01] | -.002 [-.01, .01] | -.004 [-.02, .01] | -.004 [-.01, .01] | -.005 [-.02, .004] |
| BPV x age group | .10 [-.30, .50] | .02 [-.42, .47] | .11 [-.34, .55] | .10 [-.30, .50] | .13 [-.26, .52] |
| R ACC |  |  |  |  |  |
| BPV x age | -.005 [-.02, .01] | -.001 [-.01, .01] | -.002 [-.01, .01] | -.005 [-.02, .01] | -.007 [-.02, .004] |
| BPV x age group | .15 [-.31, .61] | -.06 [-.54, .41] | .03 [-.44, .49] | .18 [-.28, .64] | .23 [-.20, .67] |

Standardized beta (ß) and 95% confidence intervals shown unless otherwise indicated.

Bolded items indicate significant systolic BPV x age/age group interaction on regional Z-ALFF.

Abbreviations: L = left hemisphere: R = right hemisphere; WB = whole brain; HC = hippocampus; PHG = parahippocampal gyrus; EC = entorhinal cortex; PC = perirhinal cortex; AM = amygdala; IN = insular cortex; ACC = anterior cingulate cortex; ALFF = amplitude of low frequency fluctuations

**Supplementary Table 4.**

Model estimates of mean systolic BP predicting regional Z-ALFF in older adults.

|  | **ALFF**  **(.01 – .10 Hz)** | **Slow2**  **(.198 - .25 Hz)** | **Slow3**  **(.073 - .198 Hz)** | **Slow4**  **(.027 - .073 Hz)** | **Slow5**  **(.01 - .027 Hz)** |
| --- | --- | --- | --- | --- | --- |
| **Region** |  |  |  |  |  |
| L WB | -.03 [-.34, .28] | .11 [-.16, .37] | .13 [-.17, .44] | -.04 [-.35, .26] | -.02 [-.32, .28] |
| R WB | -.06 [-.32, .21] | -.08 [-.37, .22] | -.17 [-.44, .10] | -.05 [-.32, .22] | -.08 [-.36, .19] |
| L HC | .15 [-.15, .45] | .002 [-.30, .30] | -.01 [-.32, .30] | .16 [-.14, .46] | .07 [-.22, .36] |
| R HC | .17 [-.12, .46] | -.06 [-.33, .22] | -.13 [-.44, .17] | .20 [-.10, .50] | .12 [-.18, .43] |
| L PHG | .03 [-.25, .31] | -.10 [-.40, .19] | -.27 [-.54, .005] | .05 [-.23, .34] | .02 [-.25, .29] |
| R PHG | -.10 [-.42, .23] | -.21 [-.52, .09] | -.33 [-.66, .007] | -.05 [-.37, .27] | -.13 [-.45, .18] |
| L EC | .10 [-.23, .43] | -.08 [-.41, .26] | -.10 [-.42, .23] | .12 [-.22, .45] | .14 [-.19, .47] |
| R EC | .10 [-.21, .40] | -.01 [-.29, .27] | -.08 [-.37, .22] | .09 [-.22, .41] | .07 [-.24, .38] |
| L PC | .05 [-.25, .36] | -.03 [-.36, .29] | -.13 [-.43, .16] | .07 [-.24, .38] | .02 [-.28, .32] |
| R PC | .07 [-.21, .34] | -.02 [-.31, .26] | -.11 [-.41, .19] | .07 [-.21, .34] | .03 [-.25, .31] |
| L AM | -.03 [-.35, .29] | -.07 [-.35, .22] | -.06 [-.38, .25] | -.05 [-.37, .26] | -.09 [-.41, .23] |
| R AM | .04 [-.27, .35] | -.02 [-.29, .26] | -.15 [-.47, .17] | .05 [-.25, .36] | .05 [-.28, .37] |
| L IN | .15 [-.16, .46] | .11 [-.18, .40] | .08 [-.23, .38] | .15 [-.16, .45] | .08 [-.23, .39] |
| R IN | .18 [-.14, .49] | -.07 [-.38, .25] | -.02 [-.35, .31] | .17 [-.14, .49] | .12 [-.20, .43] |
| L ACC | .12 [-.16, .40] | .03 [-.31, .36] | .10 [-.22, .41] | .11 [-.18, .39] | .05 [-.21, .30] |
| R ACC | .10 [-.17, .37] | .08 [-.22, .37] | .14 [-.16, .43] | .08 [-.20, .36] | .03 [-.23, .29] |

Standardized beta (ß) and 95% confidence intervals shown unless otherwise indicated.

Abbreviations: L = left hemisphere: R = right hemisphere; HC = hippocampus; PHG = parahippocampal gyrus; EC = entorhinal cortex; PC = perirhinal cortex; ALFF = amplitude of low frequency fluctuations; WB = whole brain; AM = amygdala; IN = insular cortex; ACC = anterior cingulate cortex

**Supplementary Table 5.**

Model estimates of mean systolic BP predicting regional Z-ALFF in younger adults.

|  | **ALFF**  **(.01 – .10 Hz)** | **Slow2**  **(.198 - .25 Hz)** | **Slow3**  **(.073 - .198 Hz)** | **Slow4**  **(.027 - .073 Hz)** | **Slow5**  **(.01 - .027 Hz)** |
| --- | --- | --- | --- | --- | --- |
| **Region** |  |  |  |  |  |
| L WB | -.03 [-.32, .27] | -.01 [-.29, .27] | .02 [-.26, .31] | -.02 [-.31, .27] | -.05 [-.35, .26] |
| R WB | -.07 [-.35, .20] | -.03 [-.30, .25] | -.04 [-.31, .22] | -.07 [-.35, .20] | -.06 [-.33, .22] |
| L HC | -.03 [-.30, .24] | .05 [-.22, .32] | -.007 [-.28, .26] | -.01 [-.28, .26] | -.06 [-.33, .21] |
| R HC | -.20 [-.51, .10] | -.06 [-.33, .20] | -.13 [-.41, .15] | -.19 [-.50, .12] | -.27 [-.57, .03] |
| L PHG | .11 [-.13, .34] | .11 [-.17, .38] | .08 [-.17, .33] | .12 [-.12, .35] | .08 [-.16, .31] |
| R PHG | .04 [-.25, .32] | .03 [-.24, .29] | .04 [-.25, .34] | .04 [-.24, .31] | -.05 [-.33, .22] |
| L EC | -.04 [-.35, .27] | .04 [-.24, .31] | .003 [-.28, .29] | -.04 [-.35, .27] | -.007 [-.31, .29] |
| R EC | -.11 [-.39, .18] | -.06 [-.30, .18] | -.11 [-.37, .15] | -.08 [-.37, .20] | -.10 [-.40, .19] |
| L PC | -.10 [-.39, .19] | .08 [-.20, .36] | .02 [-.26, .30] | -.11 [-.40, .19] | -.15 [-.44, .15] |
| R PC | -.21 [-.50, .09] | -.11 [-.36, .13] | -.15 [-.42, .13] | -.20 [-.49, .10] | -.24 [-.52, .05] |
| L AM | .03 [-.26, .35] | .11 [-.16, .38] | .12 [-.14, .38] | .03 [-.27, .34] | -.05 [-.35, .26] |
| R AM | -.07 [-.34, .20] | .13 [-.13, .39] | .07 [-.19, .34] | -.09 [-.37, .18] | -.15 [-.40, .11] |
| L IN | -.04 [-.27, .19] | .03 [-.25, .30] | .06 [-.18, .30] | -.06 [-.29, .18] | -.11 [-.35, .12] |
| R IN | .06 [-.22, .34] | .16 [-.08, .40] | .20 [-.03, .44] | .03 [-.26, .32] | -.07 [-.37, .22] |
| L ACC | .05 [-.21, .32] | .07 [-.21, .35] | .10 [-.19, .38] | .05 [-.21, .31] | -.03 [-.31, .25] |
| R ACC | -.01 [-.34, .33] | .06 [-.27, .39] | .02 [-.30, .35] | .01 [-.33, .34] | -.12 [-.44, .20] |

Standardized beta (ß) and 95% confidence intervals shown unless otherwise indicated.

Abbreviations: L = left hemisphere: R = right hemisphere; HC = hippocampus; PHG = parahippocampal gyrus; EC = entorhinal cortex; PC = perirhinal cortex; ALFF = amplitude of low frequency fluctuations; WB = whole brain; AM = amygdala; IN = insular cortex; ACC = anterior cingulate cortex

**Supplementary Table 6.**

Model estimates of systolic BPV predicting regional Z-ALFF in sensitivity analyses controlling for additional covariates.

|  | **ALFF**  **(.01 – .10 Hz)** | **Slow2**  **(.198 - .25 Hz)** | **Slow3**  **(.073 - .198 Hz)** | **Slow4**  **(.027 - .073 Hz)** | **Slow5**  **(.01 - .027 Hz)** |
| --- | --- | --- | --- | --- | --- |
| **Older adults** |  |  |  |  |  |
| **Additional covariate** |  |  |  |  |  |
| R HC |  |  |  |  |  |
| BP meds | **-.40 [-.68, -.12]** | **-.36 [-.61, -.12]** | **-.44 [-.72, -.16]** | **-.39 [-.68, -.09]** | -- |
| Statin | **-.36 [-.67, -.06]** | **-.32 [-.62, -.01]** | **-.40 [-.74, -.05]** | **-.35 [-.66, -.04]** | -- |
| Diabetes meds | **-.38 [-.66, -.10]** | **-.28 [-.54, -.02]** | **-.34 [-.63, -.05]** | **-.37 [-.66, -.09]** | -- |
| Psych meds | **-.38 [-.66, -.11]** | **-.29 [-.55, -.03]** | **-.35 [-.64, -.05]** | **-.38 [-.66, -.09]** | -- |
| Smoking history | **-.38 [-.65, -.11]** | **-.29 [-.55, -.03]** | **-.34 [-.64, -.05]** | **-.37 [-.66, -.09]** | -- |
| Alcohol history | **-.40 [-.66, -.14]** | **-.30 [-.55, -.05]** | **-.36 [-.64, -.08]** | **-.39 [-.66, -.12]** | -- |
| Caffeine | **-.39 [-.66, -.12]** | **-.30 [-.55, -.05]** | **-.35 [-.64, -.06]** | **-.38 [-.66, -.10]** | -- |
| Stress | **-.37 [-.64, -.09]** | **-.28 [-.54, -.02]** | **-.33 [-.62, -.04]** | **-.36 [-.64, -.08]** | -- |
| Mean BP | **-.36 [-.64, -.08]** | **-.31 [-.58, -.05]** | **-.39 [-.68, -.10]** | **-.35 [-.63, -.06]** | -- |
| Heart rate | **-.40 [-.67, -.13]** | **-.30 [-.56, -.03]** | **-.38 [-.66, -.09]** | **-.39 [-.67, -.11]** | -- |
| RMSSD | **-.37 [-.65, -.10]** | **-.31 [-.57, -.06]** | **-.37 [-.65, -.06]** | **-.36 [-.64, -.07]** | -- |
| R PHG |  |  |  |  |  |
| BP meds | **-.41 [-.72, -.09]** | **-.44 [-.72, -.16]** | -- | **-.39 [-.70, -.08]** | -- |
| Statin | -.30 [-.66, .07] | -.32 [-.67, -.02] | -- | -.29 [-.64, .06] | -- |
| Diabetes meds | **-.33 [-.64, -.01]** | **-.33 [-.63, -.03]** | -- | **-.33 [-.64, -.02]** | -- |
| Psych meds | **-.34 [-.64, -.03]** | **-.34 [-.63, -.05]** | **--** | **-.34 [-.64, -.03]** | -- |
| Smoking history | **-.33 [-.64, -.02]** | **-.34 [-.64, -.04]** | -- | **-.33 [-.63, -.03]** | -- |
| Alcohol history | **-.34 [-.65, -.03]** | **-.35 [-.64, -.06]** | -- | **-.34 [-.65, -.04]** | -- |
| Caffeine | **-.33 [-.65, -.01]** | **-.34 [-.64, -.04]** | -- | **-.33 [-.64, -.02]** | -- |
| Stress | **-.33 [-.65, -.01]** | **-.33 [-.63, -.03]** | -- | **-.33 [-.64, -.02]** | -- |
| Mean BP | **-.37 [-.68, -.05]** | **-.40 [-.69, -.01]** | -- | **-.36 [-.67, -.04]** | -- |
| Heart rate | **-.36 [-.67, -.05]** | **-.34 [-.64, -.03]** | -- | **-.36 [-.66, -.06]** | -- |
| RMSSD | **-.35 [-.66, -.03]** | **-.37 [-.66, -.08]** | -- | **-.35 [-.65, -.04]** | -- |
| L EC |  |  |  |  |  |
| BP meds | -- | **-.35 [-.68, -.01]** | -- | -- | -- |
| Statin | -- | -.33 [-.72, .07] | -- | -- | -- |
| Diabetes meds | -- | -.32 [-.64, .01] | -- | -- | -- |
| Psych meds | -- | -.32 [-.64, .002] | -- | -- | -- |
| Smoking history | -- | **-.32 [-.64, -.003]** | -- | -- | -- |
| Alcohol history | -- | -.32 [-.65, .01] | -- | -- | -- |
| Caffeine | -- | **-.34 [-.66, -.02]** | -- | -- | -- |
| Stress | -- | -.30 [-.62, .02] | -- | -- | -- |
| Mean BP | -- | **-.36 [-.69, -.03]** | -- | -- | -- |
| Heart rate | -- | **-.33 [-.66, -.002]** | -- | -- | -- |
| RMSSD | -- | -.32 [-.65, .01] | -- | -- | -- |
| R EC |  |  |  |  |  |
| BP meds | -- | **-.28 [-.56, -.001]** | -- | -- | -- |
| Statin | -- | -.31 [-.64, .01] | -- | -- | -- |
| Diabetes meds | -- | **-.28 [-.55, -.01]** | -- | -- | -- |
| Psych meds | -- | **-.28 [-.55, -.02]** | -- | -- | -- |
| Smoking history | -- | **-.29 [-.56, -.01]** | -- | -- | -- |
| Alcohol history | -- | **-.28 [-.55, -.01]** | -- | -- | -- |
| Caffeine | -- | **-.30 [-.56, -.03]** | -- | -- | -- |
| Stress | -- | **-.28 [-.55, -.004]** | -- | -- | -- |
| Mean BP | -- | **-.30 [-.58, -.02]** | -- | -- | -- |
| Heart rate | -- | **-.29 [-.56, -.01]** | -- | -- | -- |
| RMSSD | -- | **-.31 [-.58, -.05]** | -- | -- | -- |
| R PC |  |  |  |  |  |
| BP meds | -- | **-.34 [-.61, -.06]** | **-.39 [-.68, -.10]** | -- | -- |
| Statin | -- | -.28 [-.59, .03] | **-.36 [-.70, -.02]** | -- | -- |
| Diabetes meds | -- | **-.29 [-.55, -.02]** | **-.33 [-.62, -.05]** | -- | -- |
| Psych meds | -- | **-.29 [-.57, -.02]** | **-.34 [-.62, -.05]** | -- | -- |
| Smoking history | -- | **-.30 [-.57, -.02]** | **-.34 [-.62, -.05]** | -- | -- |
| Alcohol history | -- | **-.29 [-.56, -.02]** | **-.33 [-.62, -.04]** | -- | -- |
| Caffeine | -- | **-.31 [-.57, -.04]** | **-.34 [-.63, -.06]** | -- | -- |
| Stress | -- | **-.28 [-.55, -.01]** | **-.33 [-.61, -.04]** | -- | -- |
| Mean BP | -- | **-.31 [-.59, -.04]** | **-.37 [-.66, -.09]** | -- | -- |
| Heart rate | -- | **-.29 [-.57, -.02]** | **-.35 [-.64, -.06]** | -- | -- |
| RMSSD | -- | **-.33 [-.59, -.07]** | **-.36 [-.65, -.08]** | -- | -- |
| R ACC |  |  |  |  |  |
| BP meds | -- | -- | -- | -- | -.23 [-.49, .03] |
| Statin | -- | -- | -- | -- | -.28 [-.58, .02] |
| Diabetes meds | -- | -- | -- | -- | **-.27 [-.52, -.02]** |
| Psych meds | -- | -- | -- | -- | **-.26 [-.52, -.01]** |
| Smoking history | -- | -- | -- | -- | **-.26 [-.52, -.01]** |
| Alcohol history | -- | -- | -- | -- | **-.27 [-.52, -.02]** |
| Caffeine | -- | -- | -- | -- | **-.26 [-.51, -.01]** |
| Stress | -- | -- | -- | -- | -.24 [-.49, .003] |
| Mean BP | -- | -- | -- | -- | **-.27 [-.53, -.01]** |
| Heart rate | -- | -- | -- | -- | **-.28 [-.53, -.02]** |
| RMSSD | -- | -- | -- | -- | -.25 [-.51, .002] |
| **Younger adults** |  |  |  |  |  |
| **Additional covariate** |  |  |  |  |  |
| L HC |  |  |  |  |  |
| Psych meds | **-.29 [-.55, -.04]** | -- | **-.27 [-.54, -.01]** | **-.30 [-.55, -.05]** | -- |
| Smoking history | -.24 [-.51, .02] | -- | -.23 [-.49, .03] | -.25 [-.51, .01] | -- |
| Alcohol history | **-.30 [-.56, -.03]** | -- | **-.28 [-.55, -.02]** | **-.30 [-.56, -.04]** | -- |
| Caffeine | **-.30 [-.56, -.04]** | -- | **-.28 [-.54, -.01]** | **-.31 [-.56, -.05]** | -- |
| Stress | **-.33 [-.59, -.07]** | -- | **-.34 [-.59, -.09]** | **-.33 [-.59, -.07]** | -- |
| Mean BP | **-.28 [-.55, -.01]** | -- | **-.27 [-.53, -.01]** | **-.28 [-.55, -.02]** | -- |
| Heart rate | -.26 [-.52, .01] | -- | -.25 [-.50, .01] | **-.26 [-.52, -.003]** | -- |
| RMSSD | -.23 [-.49, .03] | -- | -.21 [-.45, .03] | -.24 [-.50, .01] | -- |
| R HC |  |  |  |  |  |
| Psych meds | **-.32 [-.62, -.01]** | -- | **-.30 [-.57, -.02]** | **-.32 [-.63, -.01]** | -- |
| Smoking history | -.27 [-.57, .04] | -- | -.26 [-.54, .01] | -.27 [-.58, .04] | -- |
| Alcohol history | **-.33 [-.64, -.02]** | -- | **-.32 [-.60, -.05]** | **-.33 [-.64, -.02]** | -- |
| Caffeine | **-.35 [-.64, -.06]** | -- | **-.33 [-.60, -.06]** | **-.35 [-.64, -.06]** | -- |
| Stress | **-.36 [-.67, -.06]** | -- | **-.35 [-.63, -.08]** | **-.36 [-.67, -.05]** | -- |
| Mean BP | **-.33 [-.63, -.03]** | -- | **-.31 [-.59, -.04]** | **-.32 [-.63, -.02]** | -- |
| Heart rate | **-.30 [-.60, -.01]** | -- | **-.29 [-.55, -.03]** | -.30 [-.61, .002] | -- |
| RMSSD | -.28 [-.59, .02] | -- | -.25 [-.51, .003] | -.29 [-.60, .03] | -- |
| L PHG |  |  |  |  |  |
| Psych meds | **-.25 [-.48, -.02]** | -- | -- | **-.25 [-.48, -.02]** | -- |
| Smoking history | -.22 [-.46, .01] | -- | -- | -.23 [-.46, .01] | -- |
| Alcohol history | **-.23 [-.47, -.001]** | -- | -- | **-.24 [-.47, -.01]** | -- |
| Caffeine | **-.25 [-.48, -.02]** | -- | -- | **-.25 [-.48, -.03]** | -- |
| Stress | **-.29 [-.52, -.06]** | -- | -- | **-.29 [-.52, -.07]** | -- |
| Mean BP | **-.23 [-.46, -.001]** | -- | -- | **-.23 [-.46, -.01]** | -- |
| Heart rate | -.21 [-.49, .08] | -- | -- | -.21 [-.49, .08] | -- |
| RMSSD | -.20 [-.49, .10] | -- | -- | -.20 [-.50, .10] | -- |
| L AM |  |  |  |  |  |
| Psych meds | -- | -- | **-.31 [-.56, -.05]** | -- | -- |
| Smoking history | -- | -- | **-.28 [-.54, -.02]** | -- | -- |
| Alcohol history | -- | -- | **-.30 [-.56, -.05]** | -- | -- |
| Caffeine | -- | -- | **-.32 [-.56, -.07]** | -- | -- |
| Stress | -- | -- | **-.35 [-.60, -.11]** | -- | -- |
| Mean BP | -- | -- | **-.29 [-.54, -.04]** | -- | -- |
| Heart rate | -- | -- | **-.28 [-.53, -.03]** | -- | -- |
| RMSSD | -- | -- | **-.26 [-.51, -.01]** | -- | -- |
| R AM |  |  |  |  |  |
| Psych meds | **-.27 [-.54, -.003]** | **-.30 [-.55, -.05]** | **-.33 [-.58, -.08]** | -- | -- |
| Smoking history | -.23 [-.50, .03] | **-.32 [-.58, -.07]** | **-.33 [-.58, -.07]** | -- | -- |
| Alcohol history | **-.29 [-.55, -.02]** | **-.31 [-.57, -.06]** | **-.34 [-.60, -.09]** | -- | -- |
| Caffeine | **-.30 [-.55, -.05]** | **-.33 [-.57, -.09]** | **-.35 [-.60, -.11]** | -- | -- |
| Stress | **-.31 [-.58, -.04]** | **-.33 [-.59, -.08]** | **-.36 [-.61, -.10]** | -- | -- |
| Mean BP | **-.28 [-.54, -.01]** | **-.30 [-.55, -.05]** | **-.33 [-.58, -.08]** | -- | -- |
| Heart rate | **-.27 [-.53, -.01]** | **-.30 [-.55, -.05]** | **-.33 [-.58, -.08]** | -- | -- |
| RMSSD | -.25 [-.52, .02] | **-.28 [-.54, -.03]** | **-.31 [-.56, -.06]** | -- | -- |

Standardized beta (ß) and 95% confidence intervals shown unless otherwise indicated.

Bolded items indicate systolic BPV is significantly associated with regional Z-ALFF.

Sensitivity analyses were performed in regions/frequencies found to be significant in main analyses; therefore, some column entries may be blank.

Models covaried for age, sex and the individual additional covariate.

Abbreviations: WB = whole brain; HC = hippocampus; PHG = parahippocampal gyrus; EC = entorhinal cortex; PC = perirhinal cortex; AM = amygdala; IN = insular cortex; ACC = anterior cingulate cortex; ALFF = amplitude of low frequency fluctuations; BP = blood pressure; L = left hemisphere; R = right hemisphere; RMSSD = root mean square of successive differences between normal heartbeats

**Supplementary Table 7.**

Model estimates of systolic BPV predicting Z-ALFF in anterior and posterior insula.

|  | **ALFF**  **(.01 – .10 Hz)** | **Slow2**  **(.198 - .25 Hz)** | **Slow3**  **(.073 - .198 Hz)** | **Slow4**  **(.027 - .073 Hz)** | **Slow5**  **(.01 - .027 Hz)** |
| --- | --- | --- | --- | --- | --- |
| **Older adults** |  |  |  |  |  |
| **Anterior** |  |  |  |  |  |
| L IN | .04 [-.26, .34] | -.09 [-.35, .18] | -.01 [-.32, .29] | .03 [-.27, .33] | -.03 [-.33, .28] |
| R IN | -.13 [-.44, .18] | -.10 [-.39, .20] | -.10 [-.42, .23] | -.14 [-.45, .17] | -.15 [-.46, .16] |
| **Posterior** |  |  |  |  |  |
| L IN | .02 [-.32, .35] | -.12 [-.44, .20] | -.05 [-.37, .27] | .01 [-.32, .35] | -.02 [-.36, .32] |
| R IN | -.19 [-.51, .12] | -.01 [-.36, .33] | -.06 [-.40, .28] | -.21 [-.52, .10] | -.16 [-.47, .15] |
| **Younger adults** |  |  |  |  |  |
| **Anterior** |  |  |  |  |  |
| L IN | -.04 [-.30, .23] | -.16 [-.43, .11] | -.11 [-.36, .14] | -.04 [-.31, .23] | -.004 [-.28, .27] |
| R IN | -.21 [-.49, .08] | -.20 [-.45, .05] | -.19 [-.43, .04] | -.20 [-.50, .09] | -.17 [-.47, .12] |
| **Posterior** |  |  |  |  |  |
| L IN | -.15 [-.35, .06] | -.21 [-.49, .08] | -.23 [-.47, .004] | -.13 [-.34, .07] | -.16 [-.36, .04] |
| R IN | **-.29 [-.55, -.03]** | -.14 [-.38, .10] | -.23 [-.47, .003] | **-.29 [-.55, -.03]** | **-.29 [-.56, -.01]** |

Standardized beta (ß) and 95% confidence intervals shown unless otherwise indicated.

Bolded items indicate systolic BPV is significantly associated with regional Z-ALFF.

Models covaried for age and sex.

Abbreviations: L = left hemisphere: R = right hemisphere; ALFF = amplitude of low frequency fluctuations; IN = insular cortex
